# Correction: Network proteomics of the Lewy body dementia brain reveals presynaptic signatures distinct from Alzheimer’s disease

**DOI:** 10.1186/s13024-024-00765-1

**Published:** 2024-10-11

**Authors:** Anantharaman Shantaraman, Eric B. Dammer, Obiadada Ugochukwu, Duc M. Duong, Luming Yin, E. Kathleen Carter, Marla Gearing, Alice Chen-Plotkin, Edward B. Lee, John Q. Trojanowski, David A. Bennett, James J. Lah, Allan I. Levey, Nicholas T. Seyfried, Lenora Higginbotham

**Affiliations:** 1 Center for Neurodegenerative Disease, Emory University School of Medicine, Atlanta, GA USA; 2 Department of Biochemistry, Emory University School of Medicine, Atlanta, GA USA; 3 Department of Neurology, Emory University School of Medicine, Atlanta, GA USA; 4 Department of Pathology and Laboratory Medicine, Emory University School of Medicine, Atlanta, GA USA; 5 Department of Neurology, Perelman School of Medicine at the University of Pennsylvania, Philadelphia, PA USA; 6 Department of Pathology and Laboratory Medicine, Perelman School of Medicine at the University of Pennsylvania, Philadelphia, PA USA; 7 Rush Alzheimer’s Disease Center, Rush University Medical Center, Chicago, IL USA

**Molecular Neurodegeneration (2024) 19:60**

10.1186/s13024-024-00749-1

The authors mistakenly omitted two funding sources - The BrightFocus Foundation and The American Brain Foundation (both for Lenora Higginbotham - in the original article which they wish to acknowledge via this Correction article.

