# Supplementary figures and images for: Network proteomics of the Lewy body dementia brain reveals presynaptic signatures distinct from Alzheimer’s disease
Source: Mol Neurodegener. 2024 Aug 6;19:60. doi: 10.1186/s13024-024-00749-1 (PMC11302177; doi:10.1186/s13024-024-00749-1)

Figure S1

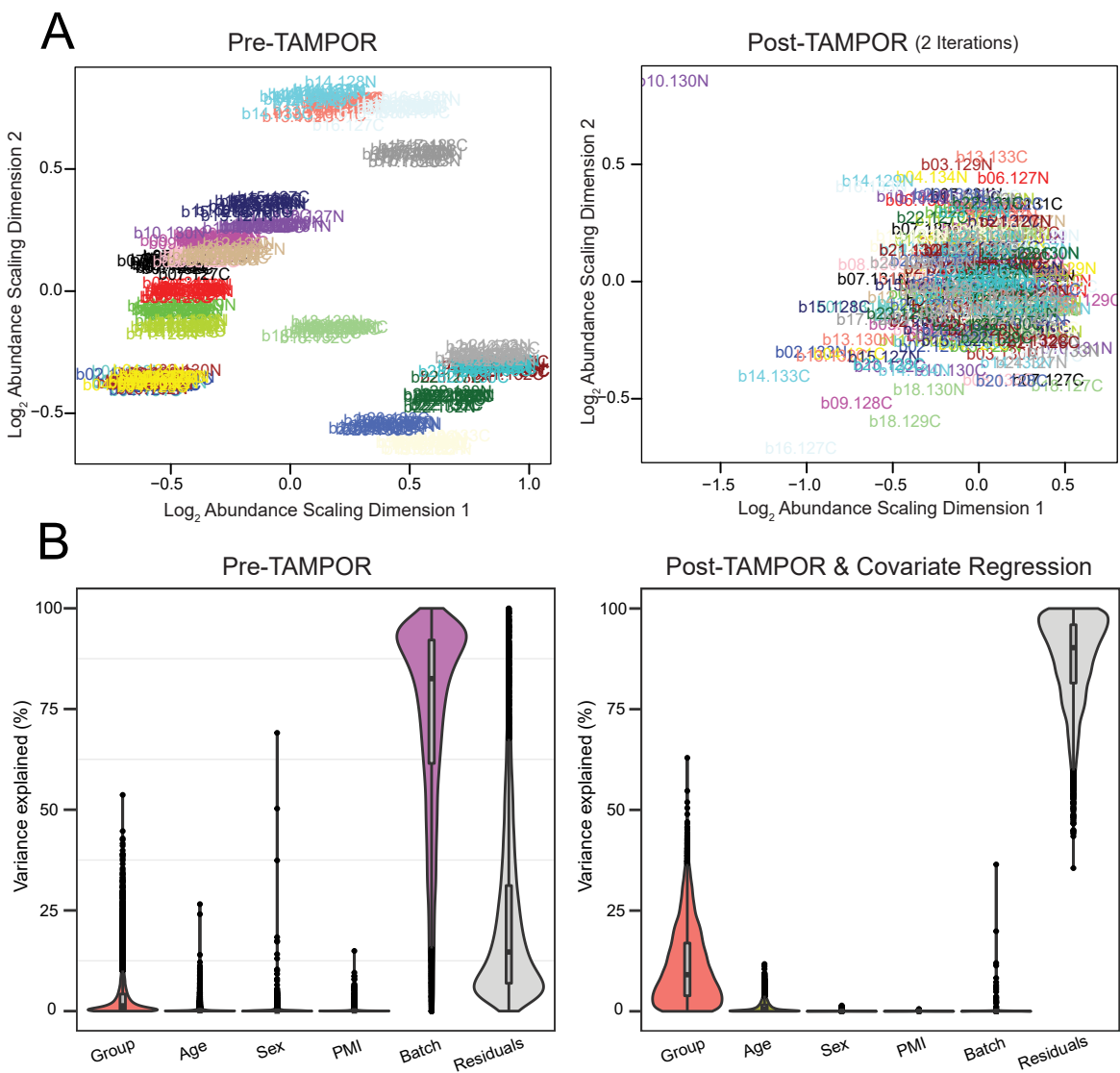

Supplement: Supplementary file 1 — Supplementary Material 1: Figure S1. TAMPOR and covariate regression of UPenn TMT-MS dataset.Multidimensional scaling plot displaying case distribution based on inter-sample variance pre- and post-TAMPOR normalization between batches in the UPenn TMT-MS dataset. This batch correction was performed across all 354 cases and 24 batches in the original UPenn cohort prior to LB subset analyses.Variance partition plots for the UPenn TMT-MS dataset pre- and post-TAMPOR and covariate regression, demonstrating that protein abundance variance due to age, sex, and post-mortem interval was effectively minimized in favor of highlighting alterations driven by group, i.e. disease diagnosis. Abbreviations: TAMPOR, tunable median polish of ratio; PMI, post-mortem interval. [file 13024_2024_749_MOESM1_ESM.pdf]

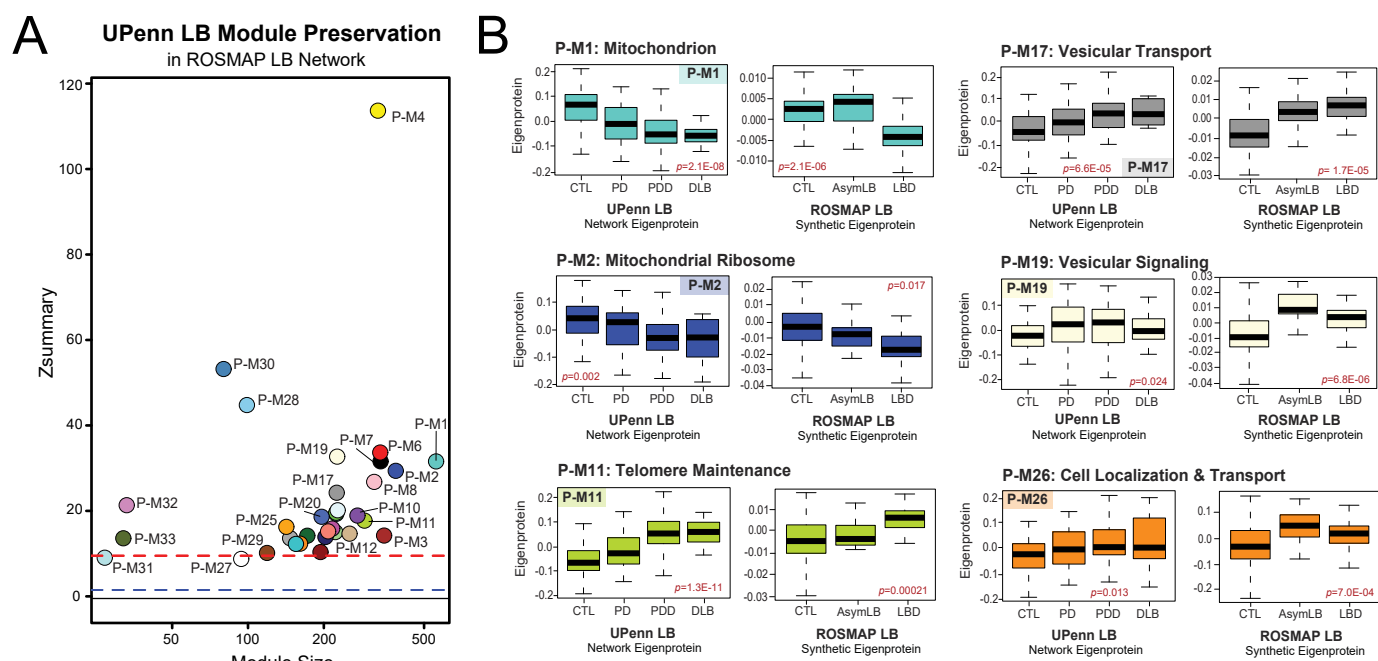

Supplement: Supplementary file 3 — Supplementary Material 3: Figure S3. LBD-associated network alterations are replicated in a ROSMAP tissue cohort. (A) Module preservation analysis of UPenn network into the ROSMAP network. Modules with a Z summary score of greater than or equal to 1.96 ( q = 0.05, blue dotted line) were considered preserved, while modules with Z summary scores of greater than or equal to 10 ( q = 1.0E-23, red dotted line) were considered highly preserved. (B) Select UPenn LBD network module eigenproteins associated with their corresponding synthetic eigenproteins in the ROSMAP network. The ROSMAP synthetic eigenproteins reflected the weighted module abundance of the top 20% of proteins by kME comprising each LBD module. ANOVA p values are provided for each eigenprotein plot. Box plots represent the median and 25th and 75th percentiles, while data points up to 1.5 times the interquartile range from the box hinge define the extent of error bar whiskers. Abbreviations: CTL, control; PD, Parkinson’s disease; PDD, Parkinson’s disease dementia; DLB, Dementia with Lewy bodies; AsymLB, Asymptomatic Lewy body pathology; LBD, Lewy body dementia. [file 13024_2024_749_MOESM3_ESM.pdf]
