# Supplementary material for: Network proteomics of the Lewy body dementia brain reveals presynaptic signatures distinct from Alzheimer’s disease
Source: Mol Neurodegener. 2024 Aug 6;19:60. doi: 10.1186/s13024-024-00749-1 (PMC11302177; doi:10.1186/s13024-024-00749-1)
Supplement: Supplementary file 2 — Supplementary Material 2: Figure S2. Module overlap between UPenn and Emory co-expression networks. A hypergeometric Fisher’s exact test was used to determine which modules shared significant overlap of protein members between the UPenn and Emory networks. The 33 modules in the UPenn network (y-axis) were aligned to the 39 modules in the Emory network (x-axis). Numbers indicate the -log10 p -value with red shading indicating the degree of significance of overlap (*, p < 0.05; **, p < 0.01; ***, p < 0.001). The green box indicates those UPenn modules that overlapped most strongly with E-24, which stood out in the Emory network given its association with synaptic ontologies, robust elevations in LBD, and selectively positive correlation to LB deposition relative to amyloid and tau. Its overlapping UPenn modules included P-M17 and P-M26, which were also SNCA-associated synaptic modules with strong increases in LBD. [file 13024_2024_749_MOESM2_ESM.pdf]

Figure S2

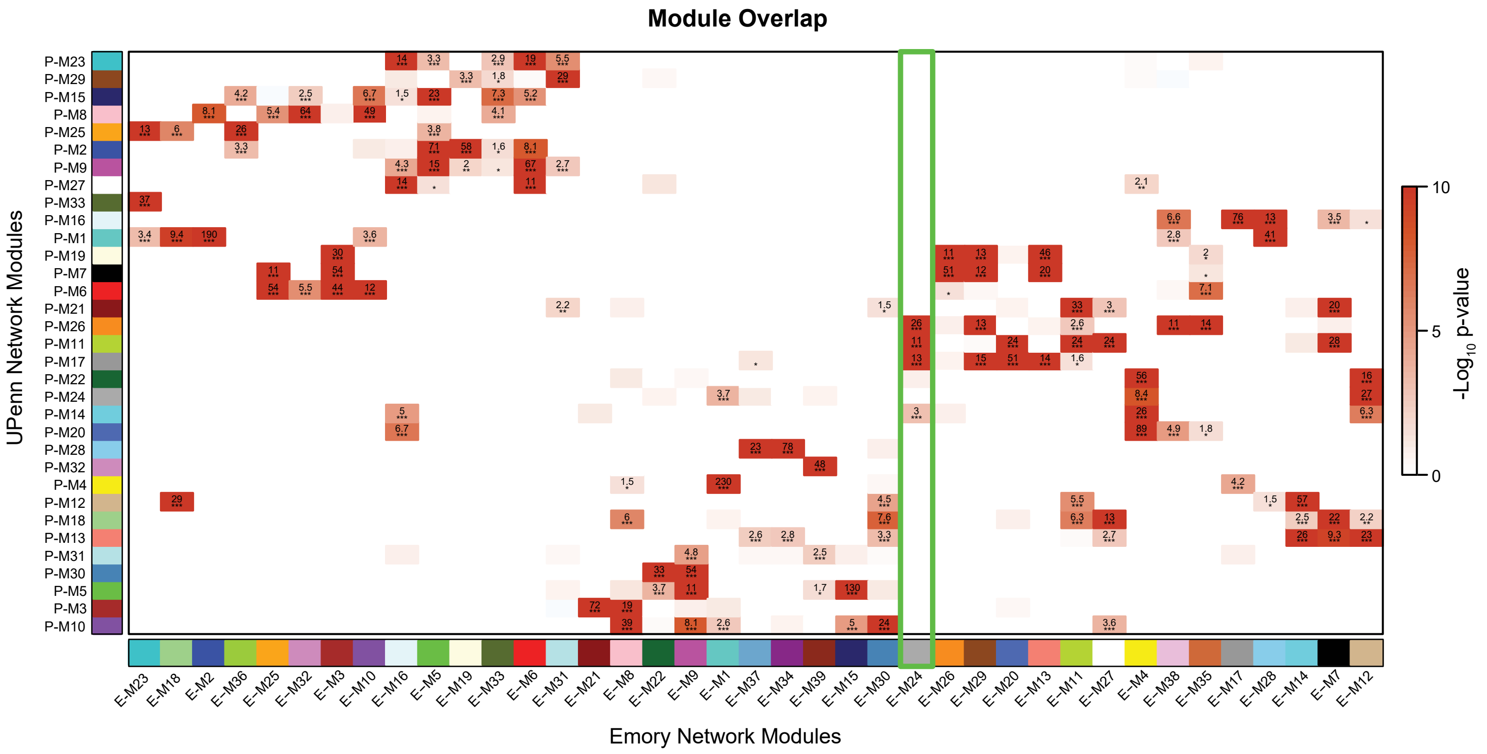
Module Overlap
UPenn Network Modules
Emory Network Modules
-Log10 p-value
10
5
0
P-M23
P-M29
P-M15
P-M8
P-M25
P-M2
P-M9
P-M27
P-M33
P-M16
P-M1
P-M19
P-M7
P-M6
P-M21
P-M26
P-M11
P-M17
P-M22
P-M24
P-M14
P-M20
P-M28
P-M32
P-M4
P-M12
P-M18
P-M13
P-M31
P-M30
P-M5
P-M3
P-M10
E-M23
E-M18
E-M2
E-M36
E-M25
E-M32
E-M3
E-M10
E-M16
E-M5
E-M19
E-M33
E-M6
E-M31
E-M21
E-M8
E-M22
E-M9
E-M1
E-M37
E-M34
E-M39
E-M15
E-M30
E-M24
E-M26
E-M29
E-M20
E-M13
E-M11
E-M27
E-M4
E-M38
E-M35
E-M17
E-M28
E-M14
E-M7
E-M12
